# Perspectivas de uso de células-tronco em cirurgia vascular

**DOI:** 10.1590/1677-5449.006516

**Published:** 2016

**Authors:** Matheus Bertanha

**Affiliations:** 1 Universidade Estadual Paulista – UNESP, Faculdade de Medicina de Botucatu, Departamento de Cirurgia e Ortopedia, São Paulo, SP, Brasil.

As células-tronco mesenquimais (CTMs) são células-tronco adultas normalmente presentes em quantidades variadas em quase todos os tecidos de origem mesodermal do organismo^1^. Essas células preservam sua capacidade multipotencial, ou seja, de se diferenciar em quase todos os tipos celulares existentes^2^. Sabe-se que elas têm a função de promover a reparação de tecidos e órgãos quando danificados, sendo esse papel crucial para o controle da homeostasia tecidual (com a realização de forma equilibrada da substituição das células senescentes)^3^. Levando-se em consideração a orientação da International Society for Cellular Therapy (ISCT)^4^, há necessidade de se comprovar a identidade das células para que se possa afirmar que elas são realmente CTMs. Isso se faz com a observação de pelo menos três diferentes características: 1) capacidade das células de aderir ao plástico do frasco de cultura e proliferar rapidamente, com a formação de colônias celulares e a apresentação de aspecto morfológico semelhante ao dos fibroblastos; 2) capacidade de se diferenciar em pelo menos três linhagens celulares distintas quando submetidas a estímulos específicos (por exemplo, fatores de crescimento), basicamente em tecido cartilaginoso, tecido ósseo e tecido adiposo; e 3) manutenção de perfil fenotípico clássico quando analisadas por técnica de citometria de fluxo, apresentando expressão positiva para alguns marcadores de superfície celular, como CD73, CD90 e CD105, e expressão negativa para CD11b, CD14, CD19, CD34, CD45, CD79 e HLA-DR (*cluster* de diferenciação – CD, do inglês, *cluster of differentiation*). Esses três critérios são suficientes para a caracterização das CTMs, mas um teste de perfil genômico pode ser usado em substituição^4^.

Quanto à origem, as CTMs adultas podem ser classificadas como: 1) hematopoiéticas, que formam células sanguíneas; e 2) estromais/mesenquimais, que podem se diferenciar em quase todos os outros tecidos não hematopoiéticos^5^. As CTMs são normalmente obtidas em grande número nos tecidos onde estão presentes em maior quantidade e/ou onde há maior facilidade para sua coleta, como o sangue da medula óssea, o tecido adiposo e o cordão umbilical^6^. Sua concentração pode variar de acordo com a idade e o local de obtenção, representando 0,1% ou menos da fração mononuclear quando se utiliza a técnica de punção aspirativa de medula óssea em seres humanos adultos^7^. As CTMs também podem ser obtidas por recuperação hematopoiética a partir de aférese de sangue periférico, sendo potencialmente ampliadas em número após sua mobilização por hormônios de crescimento específicos (fator estimulador de colônias de granulócitos – G-CSF)^1^.

Diante da diversidade de situações em que a medicina atual apresenta limitações, a aplicação da engenharia celular poderá ser uma alternativa viável na terapêutica regenerativa ou substitutiva de tecidos. Nesse contexto, essas técnicas são consideradas novos domínios da medicina translacional, envolvendo a terapia celular e a engenharia de tecidos, que despontam como propostas inovadoras dentro de um panorama no qual a medicina será aplicada de forma direcionada ao doente^8^.

Com o envelhecimento natural da população mundial, há um crescente número de pessoas acometidas por doenças crônicas, entre elas os problemas de origem cardiovascular, que figuram como a primeira causa de mortalidade da população ocidental adulta. A doença aterosclerótica é a principal vilã do sistema cardiovascular e, muitas vezes, tem complexa abordagem terapêutica. O advento da cirurgia endovascular lançou uma nova perspectiva para o tratamento desses doentes, com procedimentos menos invasivos e menores taxas de morbimortalidade, mudando conceitos de tratamento previamente estabelecidos.

Entretanto, há uma parcela de pacientes com doença arterial periférica e isquemia crítica de membros inferiores em que não se consegue êxito com técnicas endovasculares e/ou que não são elegíveis para o tratamento convencional com pontes (*bypass*), restando como alternativa apenas a amputação do membro acometido. Particularmente, terapias alternativas com CTM e engenharia de tecidos podem ser aplicadas nesses casos. Isso pode ocorrer em algumas situações extremas, quando: 1) não há uma veia autóloga adequada para a confecção da ponte; 2) não se pode utilizar uma prótese sintética como o politetrafluoreto expandido (PTFE) ou o dácron em decorrência de infecção local ou sistêmica; 3) não há um deságue arterial adequado (*outflow*); 4) há necessidade de derivações longas para artérias infrapatelares na ausência de veia autóloga adequada^9^; 5) artérias são de pequeno calibre para receber uma ponte distal com incompatibilidade de calibre. Esses casos possivelmente se beneficiariam da engenharia de tecidos ou mesmo da terapia celular.

Dessa forma, podem-se considerar como possíveis formas futuras de emprego das CTMs em cirurgia vascular, entre outras: 1) engenharia de vasos sanguíneos, em que se produziria um vaso sanguíneo com células autólogas e com características de comprimento e espessura específicas para revascularização arterial por ponte. Tal processo poderá ser viabilizado pela engenharia de tecidos. Será necessário que um arcabouço tubular receba as CTMs, e que estas sejam estimuladas à diferenciação nos tipos celulares mais importantes para o vaso sanguíneo (endotélio e músculo liso)^10^; 2) estímulo à angiogênese por meio de semeadura das CTMs através de micropunções para o tratamento de isquemia de membros inferiores. A atuação dessa forma de terapia celular com CTM pode ser tanto de forma parácrina, com atuação sobre outras células reparadoras, como de forma direta, promovendo a angiogênese e a regeneração tecidual^1^; 3) aplicação de CTM como curativo local para o tratamento de úlceras crônicas complexas. Nessa modalidade de terapia celular, as CTMs aplicadas de forma tópica podem estimular a angiogênese e a reparação tecidual^11^.

Os resultados de estudos clínicos permanecem promissores, levando-se em consideração dados apresentados em uma metanálise sobre o assunto que demonstrou redução das taxas de amputação e melhora do índice tornozelo-braquial, sem incremento de risco para os pacientes^12^. Esses estudos geralmente são realizados com CTMs de coleta direta ou apenas com separação por centrifugação, e são raros os que se utilizam de diretrizes técnicas de coleta, expansão e caracterização, conforme as orientações da ISCT. Por isso, há necessidade de estudos com esse delineamento para que haja um melhor esclarecimento da aplicação clínica de CTMs^12^.

Do ponto de vista da engenharia de tecidos, o desafio é constituir um substituto arterial biocompatível que apresente maior tolerância a infecções e que promova um ambiente propício para a regeneração tecidual. Weinberg e Bell^9^ apresentaram o primeiro protótipo de vaso sanguíneo produzido através de engenharia de tecidos. No ensaio, o vaso sanguíneo foi produzido com o implante de endotélio, músculo liso e fibroblastos na parede de um vaso bovino, o que tornou possível produzir um vaso sanguíneo completo. Entretanto, esse modelo não pôde ser aplicado cirurgicamente por sua baixa resistência mecânica. Desde então, muitos pesquisadores vêm testando modelos experimentais de vasos sanguíneos produzidos de várias maneiras, como através de arcabouços sintéticos bioabsorvíveis (colágeno purificado, ácido poliláctico – PLA, ou ácido poliglicólico – PGA), mas, mais uma vez, a resistência mecânica tem sido um limitante^10^. Como alternativa para solucionar esses problemas, têm-se buscado técnicas que promovam a junção dos conhecimentos de engenharia celular e de diferenciação das CTMs^9^.

Outros estudos em pequenas séries de casos ou relatos isolados em humanos vêm apresentando sucesso técnico na aplicação de enxertos alogênicos (de cadáveres) ou xenogênicos (derivados de animais - ovelhas ou porcos) com uso de CTMs humanas, mas muitas vezes envolvendo falhas relacionadas à resposta imunológica autoimune e consequente hiperplasia miointimal ou formação de aneurismas^10^. Olausson et al.^13^ apresentaram o resultado do uso de um enxerto alogênico de veia porta descelularizada com recelularização do enxerto com células autólogas, linfomononucleares de sangue periférico, para o tratamento de uma menina de 10 anos com obstrução portal extra-hepática. Obteve-se sucesso técnico, mas com necessidade de resgate por estenose no enxerto após um ano de seguimento, que foi tratado com angioplastia transluminal percutânea por balão com sucesso. Entretanto, ainda há necessidade de mais estudos para confirmação científica, embasada principalmente em modelos experimentais. De qualquer forma, a engenharia de tecidos e a terapia celular não são utopias, mas uma realidade muito próxima da aplicação clínica.

Em nossa instituição, uma parceria firmada entre o grupo de pesquisas com CTM do Laboratório de Engenharia Celular e a disciplina de Cirurgia Vascular vem realizando uma série de estudos em modelo animal para melhorar o desenvolvimento das técnicas de engenharia celular e vasos sanguíneos. Basicamente, temos realizado a descelularização de veias (veias cavas de coelhos) para produção de um arcabouço vascular biológico e biocompatível. Na sequência, aplicamos o que se sabe sobre CTM e diferenciação celular para construção de neovasos. Temos preferido o uso de CTMs obtidas de tecido adiposo (gordura interescapular da região dorsal do coelho) e de fatores de crescimento celular obtidos dos grânulos alfa de plaquetas, promovendo assim a diferenciação endotelial para reconstrução de vasos sanguíneos e o uso futuro para confecção de pontes arteriais. Paralelamente ao que se apresenta na literatura, nossa equipe investiga se o arcabouço obtido com a descelularização da veia terá vantagens sobre os arcabouços sintéticos, principalmente com relação à sua força mecânica^14^^,^ 15. No futuro, com a transposição da pesquisa experimental para a pesquisa aplicada em humanos, acreditamos que poderá ser mais simples a aplicação em modelos que utilizarão como base a veia safena magna. Essa veia poderá ser obtida de doadores de múltiplos órgãos e permitirá trabalhar com segmentos longos, simulando um ambiente vascular bastante natural e propício para acomodar as CTMs autólogas e promover sua diferenciação em endotélio e músculo liso. De qualquer forma, os dados apresentados em experimentos realizados em todo o mundo demonstram que o conhecimento em terapia celular e engenharia de tecidos vem caminhando de forma promissora, sendo provável que essa tecnologia esteja disponível como alternativa para casos específicos da prática clínica em médio prazo.
