# Supplementary material for: Perspectivas de uso de células-tronco em cirurgia vascular
Source: J Vasc Bras. 2016 Jul-Sep;15(3):173–5. [Article in Portuguese] doi: 10.1590/1677-5449.006516 (PMC5829752; doi:10.1590/1677-5449.006516)
Supplement: Translation

# Translation: Prospects for applications of stem cells in vascular surgery

**Authors:** Matheus Bertanha

**Affiliations:** 1 Universidade Estadual Paulista – UNESP, Faculdade de Medicina de Botucatu, Departamento de Cirurgia e Ortopedia, São Paulo, SP, Brazil.

Mesenchymal stem cells (MSCs) are adult stem cells that are normally present in varying quantities in almost all of the body’s tissues that have mesodermal origins.^1^ These cells have maintained their multipotent capacity, i.e. the capacity to differentiate into almost all of the different types of cells that exist.^2^ It is known that they function to promote repair of tissues and organs that have suffered damage and this role is crucial to control of tissue homeostasis (through balanced substitution of senescent cells).^3^ As stated in International Society for Cellular Therapy (ISCT) guidelines,^4^ it is necessary to confirm the identity of cells before it can be stated that they are true MSCs. This confirmation is based on observation of at least three different characteristics: 1) the capacity for cells to adhere to the plastic of the culture flask and proliferate rapidly, forming cell colonies and exhibiting a morphological appearance similar to fibroblasts; 2) the capacity to differentiate into at least three distinct cell lineages when subjected to specific stimuli (for example, growth factors), basically, into cartilage tissue, bone tissue, and adipose tissue; and 3) a classic phenotypical profile when analyzed by flow cytometry, with positive expression of certain cell surface markers such as CD73, CD90 and CD105, and negative expression for CD11b, CD14, CD19, CD34, CD45, CD79 and HLA-DR (cluster of differentiation – CD). These three criteria are sufficient to classify cells as MSCs, but a genome profile test can be used instead.^4^

Adult MSCs can also be classified by origin, as either 1) hematopoietic, which form blood cells, or 2) stromal/mesenchymal, which can differentiate into almost all other non-hematopoietic tissues.^5^ Mesenchymal stem cells can normally be obtained in large numbers from the tissues in which they are present in greater quantities and/or from which it is easier to collect them, such as from blood of bone marrow, adipose tissue, and umbilical cord.^6^ Their concentration can vary depending on age and site of collection and they can account for 0.1% or less of the mononuclear fraction when the needle aspiration technique is used to obtain them from bone marrow in adult humans.^7^ Mesenchymal stem cells can also be obtained by hematopoietic recovery by separation of peripheral blood and can potentially be increased in number after mobilization by specific growth hormones (granulocyte-colony stimulating factor – G-CSF).^1^

In view of the diverse range of situations in which current medicine suffers from limitations, application of cell engineering could be a viable option for treatments to regenerate or substitute tissues. Such techniques are considered new domains in translational medicine, involving cell therapy and tissue engineering, which have emerged as innovative proposals within a panorama in which medicine will be applied in a patient-directed manner.^8^

With the natural aging of the world population there is a growing number of people suffering from chronic diseases, including those of a cardiovascular origin, which are the number one cause of mortality in the adult Western population. Atherosclerotic disease is the principal culprit in the cardiovascular system and treatment approaches are very often complex. The advent of endovascular surgery opened up a new perspective for treatment of these patients, with less invasive procedures and lower rates of morbidity and mortality, changing the previously established concepts of treatment.

However, there is a set of patients with peripheral arterial disease and critical ischemia of the lower limbs for whom success cannot be achieved with endovascular techniques and/or who are not eligible for conventional treatment with bypasses, and for whom the only remaining option is amputation of the affected limb. Alternative treatments using MSCs and tissue engineering may find applications in these cases in particular. These cases can arise in a number of extreme situations, such as when: 1) there is no adequate autologous vein that can be used for a bypass; 2) a synthetic prosthesis such as expanded polytetrafluoroethylene (PTFE) or dacron cannot be used because of local or systemic infection; 3) there is insufficient arterial outflow; 4) there is a need for long shunts to infrapatellar arteries in the absence of a suitable autologous vein;^9^ or 5) arteries are of too small a caliber to receive a distal bypass with incompatible caliber. These cases could possibly benefit from tissue engineering or even cell therapy.

There are therefore, at least, two possible methods in which MSCs could be used in vascular surgery in the future. 1) Engineering of blood vessels, in which a blood vessel would be produced using autologous cells and with specific characteristics of length and thickness necessary for arterial revascularization via bypass. Such a process could be achieved by tissue engineering. A tubular scaffold would be needed to receive the MSCs, which would have to be stimulated to differentiate into the most important cell types for a blood vessel (endothelium and smooth muscle).^10^ 2) Stimulation of angiogenesis by seeding MSCs via micropuntures, for treatment of lower limb ischemia. This form of cell therapy with MSCs could work either in a paracrine manner, acting on other reparative cells, or directly, provoking angiogenesis and tissue regeneration.^1^ 3) Administration of MSCs as a local dressing for treatment of complex chronic ulcers. In this type of cell therapy, MSCs applied topically could stimulate angiogenesis and tissue repair.^11^

The results of clinical studies remain promising, considering data presented in a meta-analysis of the subject demonstrating a reduction in rates of amputation and improved ankle-brachial indexes, without increasing risk to patients.^12^ These studies are generally conducted using MSCs collected directly or with separation by centrifugation alone, and it is rare for studies to follow the technical directives for collection, expansion, and typing, as recommended by the ISCT. There is therefore a need for studies with this type of design if further light is to be shed on the clinical applications for MSCs.^12^

From the point of view of tissue engineering, the challenge is to construct a biocompatible arterial substitute that offers greater tolerance of infections and provides an environment that is suitable for tissue regeneration. Weinberg and Bell^9^ exhibited the first prototype blood vessel produced by tissue engineering. In their experiment, the blood vessel was produced by implanting endothelium, smooth muscle, and fibroblasts in the wall of a bovine vessel, which made it possible to produce a complete blood vessel. However, the model could not be used in surgical applications because of its low mechanical resistance. Since then, many researchers have been testing experimental models of blood vessels produced in a variety of ways, such as on synthetic bioabsorbable scaffolds (purified collagen, polylactic acid – PLA, or polyglycolic acid – PGA), but once again mechanical resistance has been a limiting factor.^10^ As a possible option for solving these problems, attempts have been made to develop techniques that combine the knowledge of cellular engineering with that of differentiation of MSCs.^9^

Other studies in small cases series or isolated reports in humans have described technical success using allogeneic (cadaveric) or xenogeneic (from animals – sheep or pigs) grafts together with human MSCs, but they are very often subject to failures related to autoimmune immunoresponse and consequent myointimal hyperplasia or formation of aneurysms.^10^ Olausson et al.^13^ exhibited the result of an allogeneic graft made from a decellularized portal vein that was recellularized with autologous lymphomononuclear cells from peripheral blood and used to treat a 10-year-old girl with extrahepatic portal obstruction. They achieved technical success, but after 1 year of follow-up the patient had to be treated for stenosis of the graft, which was successfully achieved with percutaneous transluminal balloon angioplasty. Notwithstanding, there is still a need for more studies for scientific confirmation, primarily based on experimental models. Nevertheless, tissue engineering and cell therapy are not utopian dreams, they are a reality that is very close to clinical application.

At our institution, a partnership between a team that researches MSC at the Cellular Engineering Laboratory and the vascular surgery department has led to a series of studies with animal models conducted to improve the level of development of techniques for engineering of cells and blood vessels. Basically, we have decellularized veins (vena cava from rabbits) to produce a biological and biocompatible vascular scaffold. We then apply what is known about MSC and cell differentiation to construct neovessels. We prefer to use MSCs obtained from adipose tissue (interscapular fat from the dorsal region of rabbits) and cellular growth factors obtained from platelet alpha granules, thereby provoking endothelial differentiation for reconstruction of blood vessels for future use in arterial bypasses. In parallel with what is being published in the literature, our team is investigating whether the scaffold obtained by decellularization of veins offers advantages over synthetic scaffolds, primarily in terms of mechanical strength.^14^^,^ 15 In the future, as the experimental research is transposed to applied research in humans, we believe that it might be simpler to apply the methods in models that will use the great saphenous vein as a basis. These veins could be obtained from multiple organ donors, which would enable us to work with long segments, simulating a very natural vascular environment that is well suited to accommodate autologous MSCs and promote their differentiation into endothelium and smooth muscle.

In all events, the data reported from experiments conducted all over the world demonstrate that knowledge is advancing in cell therapy and tissue engineering in a promising manner and it is probable that this technology will be available as an option for specific cases in clinical practice in the medium term.
